# The relationship between performance appraisal interval and employees’ proactive working behavior – analysis based on time-gain effect and time-loss effect

**DOI:** 10.3389/fpsyg.2023.1213547

**Published:** 2023-08-31

**Authors:** Jun Yang, Jun Ma, Liping Li

**Affiliations:** School of Management, Shanghai University, Shanghai, China

**Keywords:** performance appraisal interval, proactive work behavior, delay of gratification, perceived uncertainty, supervisor developmental feedback

## Abstract

**Introduction:**

Performance appraisal is the most widely motivation means for employees’ initiative and work improvement. As a large number of organizations are moving from traditional annual performance appraisal to more frequent appraisals, there is little research to compare the motivational effect of different performance appraisal intervals.

**Methods:**

We explore the relationship between performance appraisal interval (PAI) and positive work behavior (PWB) as well as how to improve the relationship between them. Drawing from the psychological momentum (PM) theory, we constructed a model of the dual effect (the time-gain effect and time-loss effect) of PAI on PWB as well as their boundary conditions.

**Results:**

A cross-level analysis of 622 employees in 57 teams indicated that: (1) PAI exerted a positive but marginal decreasing effect on delay of gratification (DG), and then increase PWB indirectly (i.e., the time-gain effect). (2) PAI exerted a positive and marginal increasing effect on perceived uncertainty (PU), and then decrease PWB indirectly (i.e., the time-loss effect). (3) According to the additive principle of the benefit and cost proposed by Hanns et al (2016), the addition of the time-gain effect and time-loss effect leads to an inverted U-shape effect of PAI on PWB. (4) Supervisor developmental feedback (SDF) moderated the inverted U-shape effect of PAI on PWB.

**Discussion:**

This research enriches the application of PM theory in performance appraisal research, advances employee proactivity research from a perspective of organizations’ time mechanisms, and also provides a theoretical basis for leaders to adopt developmental feedback as an optimization strategy.

## 1. Introduction

Under the increasing upgrade business competition, a large number of companies have moved away from traditional annual performance appraisals towards more frequent appraisals, for compressing timelines and accelerating performance improvement (Murphy et al., 2018). However, the present research focuses largely on identifying and fixing performance issues such as performance indicators (Buckingham and Goodall, 2015; DeNisi and Murphy, 2017), performance rating (Murphy, 2020), performance equity (Leung et al., 2001), and performance feedback (Atwater et al., 2007; Bracken et al., 2016). There is not a good understanding of the temporal mechanisms or cycle of performance appraisal (Shipp and Cole, 2015). With the increased work complexity and task unpredictability, performance improvement depends largely on employees’ initiative and positive behaviors, such as self-starting, problem-solving, or task revision (Parker et al., 2006; Grant et al., 2009; Parker et al., 2019). But there is little empirical evidence indicating that frequent appraisals do better than traditional ones in motivating such positive behaviors (Carl and Michael, 2017; Xu et al., 2022). Therefore, we aim to explore the relationship between performance appraisal interval and employees’ positive work behavior.

Proactive work behavior (PWB) is the self-initiated and future-oriented actions and initiative in the working process that aim for work improvement, involving behavior such as self-improvement, task revision, problem-solving, and changing the situation or even transcendent behavior (Parker et al., 2019). According to previous research, employees’ work proactivity and positive behaviors can be determined by a dual trade-off psychological process between the value, possibility, and attainment time of related rewards (O’Donnell et al., 2017; Stein et al., 2017; Wang and He, 2020). Align with this, task timeframe, as a considered factor of whether initiative actions can be achieved (possibility) and the waiting time (attainment time) of related rewards, can influence employees’ PWB (Tu and Soman, 2014). PAI is the interval between two adjacent performance appraisals, which develop the task timeframe and thus related to the perceived possibility of goal attainment as well as the delayed length of related rewards. Therefore, shortening or extending the PAI can influence their perception process of time gain and time loss and influences employees’ PWB.

Specifically, on the one hand, extending PAI conveys the information that the organization focuses on long-term orientation and future-oriented work practice (Song et al., 2023). A longer-term PAI encourages employees to disregard such “immediate but smaller” benefits and choose “delayed but larger” benefits,” that is, increase their tendency to delay of gratification (DG). DG can exert a motivational effect and promote individuals to take initiative for higher income, position, and self-development (Renn et al., 2005), indirectly increasing future-oriented behaviors and work initiative (Lin et al., 2019; Parker et al., 2019; Zhang et al., 2022). In this vein, we expected that extending PAI could increase employees’ DG and then improve their PWB indirectly. On the other hand, with the extension of PAI, unpredictable factors or uncontrollable events in the delay length increase, which makes employees perceive great risk and uncertainty of the future rewards (Wang and He, 2020), leading to individual perceived inability to predict future state accurately, that is the perceived uncertainty (PU) increase. Individuals tend to be risk-averse (Ma et al., 2023) and PU has been proven to be a main factor to decrease employees’ proactive behavior (Bordia et al., 2004a; Hon et al., 2014). We thus expect that extending PAI increases an individual’s PU and decrease their PWB indirectly. According to the above, there are two mutually antagonistic action mechanisms between PAI and PWB. According to Grant and Schwartz (2011) and Haans et al. (2016), when the increase in the independent variable increases (decreases) the dependent variable, it is named the gain (loss/cost) effect. Align with this, there is a time-gain effect and time-loss effect in our study. Since the gain and loss effects from the changing of independent variables are difficult to balance or cancel each other out, there tend to exert nonlinear effects between them (Haans et al., 2016). According to the additive principle of the benefit effect and cost effect proposed by Haans et al. (2016), we expected there may be a nonlinear relationship between PAI and PWB, that is, excessively extended PAI has an erosive effect on employees’ PWB, leading to an overall inverted U-shape effect of PAI on PWB.

We applied psychological momentum (PM) theory to integrate the paradoxical temporal effect of PAI on PWB. According to PM theory, individuals often anticipate the outcomes of their behaviors, and the perceived probability can stimulate them with a PM (positive or negative), resulting in an increased or decreased level of motivation for engaging in it (Markman and Guenther, 2007; Iso-Ahola and Dotson, 2014). Stated differently, setting outcome-related patterns or stimuli (e.g., time gap; Iso-Ahola and Dotson, 2014) can impart individuals an impetus or PM. The triggered PM will act on an individual’s cognitive experience and further influence their behavioral patterns (Markman and Guenther, 2007; Briki and Markman, 2018). Therefore, our study aims to examine the dual effects of PAI based on PM theory.

Supervisor developmental feedback (SDF) involves future-based helpful information, guidance, or planning aiming at individuals’ performance improvements and future development (Zhou, 2003). Different from general feedback which evaluates past behavior or performance, developmental feedback focuses more on assisting employees in the improvement and future development. The present research proposed that supervisors if providing guidance or information for goal attainment, current work progress, task timeline, or improvement methods, can increase employees’ future orientation and also offset their perceived uncertainty in the workplace (Zhou, 2003; Li et al., 2011). Similarly, PM theory also indicated that feedback, especially providing concrete solutions or guidance, can enhance individuals’ clarity of goal progress, and further improve their intrinsic motivation and psychological momentum (Iso-Ahola and Dotson, 2014). Therefore, we consider SDF as a moderator of the inverted U-shaped relationship.

By constructing the curvilinear relationship between PAI and PWB as well as identifying its boundary condition, our study contributes to the current literature in two ways. Firstly, while the well-documented research on the influencing factors of PWB, the effect of the organization’s temporal mechanism has been largely neglected. Although some researchers have begun to explore the influence of time factors on individual proactive behaviors, most of them focus on the subjective perception of the time factors, such as time pressure (Urbach and Weigelt, 2019), temporal categorization (Tu and Soman, 2014), and future orientation (Strauss et al., 2012). By focusing on the temporal framing effect of PAI, we changed from subject factors to objective organization timeframe, and provide a new explanatory mechanism from the time perspective for stimulating PWB. Secondly, since the “time” element becomes particularly sensitive and critical in the task situation (Tobin and Grondin, 2012; Stiglbauer, 2017), this study extends the literature on the time effectiveness of performance appraisal. It is also a useful supplement to the empirical research on performance appraisal as well as a possible perspective for further exploration. Thirdly, according to the additive principle of the benefit and cost proposed by Haans et al. (2016), we add the time-gain effect and time-loss effect of PAI on PWB together and proposed an inverted U-shaped relationship between PAI and PWB. By exploring a more comprehensive explanation of how PAI acts on employees’ PWB, we aim to verify the instability of the time-related effect proposed by Shipp and Cole (2015). Meanwhile, we found a compatible explanatory mechanism for the contradictory temporal effects. Finally, we highlight the importance of SDF in moderating the predicted curvilinear relationship between PAI and PWB and thus provide a condition about when this predicted curvilinear relationship is more positive. By introducing such leadership variables as an important optimization strategy for enhancing the motivational effectiveness of PAI, this study offers a new pathway of how SDF influences employee PWB and has implications for motivating PWB in a relatively long-term PAI.

**Figure 1:**
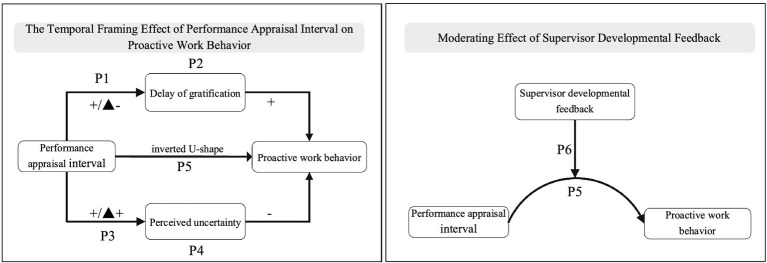
The framework of temporal framing effect of PAI on PWB.

Overall, we proposed a model of the dual temporal effect of PAI on PWB. As the mediation of DG and PU, we discuss the time-gain effect, time-gain effect, and the overall inverted U-shape relationship between PAI and PWB. Based on the moderating role of SDF, we discuss how to optimize the inverted U-shape relationship between PAI and PWB, as depicted in Figure 1.

## 2. Theoretical foundations and hypothesis development

### 2.1. The time-gain effect of PAI

#### 2.1.1. The relationship between PAI and DG

According to PM theory, outcome-related events or stimuli (e.g., score, performance, time gap; Iso-Ahola and Dotson, 2014) can impart individuals a PM. The strength of PM is influenced by the perceived value and perceived possibility of the events (Markman and Guenther, 2007). According to Reynolds and Schiffbauer (2005) and Wittmann et al. (2007), DG is determined largely by the value of the delayed reward and the perceived possibility of getting it. The perceived value of the reward would be determined by its amount and the perception of the delay length. Therefore, assessing such factors plays an important role in the success or failure of DG. Under the performance appraisal (PA) system, employees must complete performance targets within a given performance appraisal interval, and then they can get a future reward (Byron and Khazanchi, 2012; DeNisi and Smith, 2014). PAI is directly related to the value of performance rewards, the perceived possibility of attainment, and its delay length, which can change individuals’ expectations of the performance rewards and conveys to employees the organizational time preference and the timeframe of the work process. Accordingly, we assumed that PAI positively affects employees’ DG.

Specifically, long-term rewards usually have greater value (May, 2017). Compared with the short-term PAI, the longer PAI is accompanied by a larger amount of rewards. It can bring employees a greater and more continuously extended PM and keep their expectations at a long-term positive level, which improves employees’ future orientation and makes them pay more attention to long-term benefits rather than the present (Liu and Wang, 2021). Therefore, their tendency to DG can be improved. Meanwhile, with the extension of PAI, the increased time affluence can increase the possibility to achieve the exploration of the frontiers and get the outcome, and then being rewarded, which affords employees to get out of passive execution and “immediate but smaller” benefits but choosing “delayed but larger” benefits.” Besides, according to PM theory, given the dependency structure among consecutive performance phenomena, once psychological momentum is interrupted, it is more difficult to regain high-level of positive momentum as before, and the cost of recovering task progress is greater than steady progress (Markman and Guenther, 2007; Guenther and Kokotajlo, 2017). Therefore, to avoid performance interruption and internal friction, individuals will not easily break the positive state endogenous, but try to subjectively enhance persistence and self-control ability in the process of performance attainment, which is conducive to reinforcing the tendency of delayed gratification.

Since the subjective value of a reward is reduced as a function of time, the length of delay is an important factor in the delay of gratification (Wittmann et al., 2007). The reward may become unavailable over time (Stolarski et al., 2011; Macaskill et al., 2019). Similarly, PM theory has indicated that the “momentum” triggered by the initial event can only last for a limited time and then dissipate gradually with the development of the event (Iso-Ahola and Dotson, 2014). That is, as a dynamic system composed of time-related factors, the PM might display nonlinearity and show a disproportionate mutation in the development process (Briki and Markman, 2018). Aligning with this, we assume that the tendency of DG triggered by PAI does not exist continuously and uniformly. When the distance between future performance rewards and current work behavior is extending to an unacceptable level, the PM cannot be transmitted to the further future and the positive effect from PAI on DG weakens gradually. Therefore, there is a positive but marginal decreasing effect of PAI on DG.

*Hypothesis 1*: PAI has a positive but marginal decrease effect in DG.

#### 2.1.2. The mediation of DG between PAI and PWB

According to PM theory, the positive PM from the outcome-related stimuli can make them develop a set of expectations regarding the displacement of the goal, resulting in an increased level of motivation for engaging in it and further influencing their subsequent behavioral response and performance. When employees then attain a higher and long-term PM, they would become bolder and more positive, engage in positive behaviors, and usually have high performance (Iso-Ahola and Dotson, 2014; Briki and Markman, 2018). We thus assumed that DG is positively related to PWB and plays a positive mediating role between PAI and PWB.

Specifically, PWBs go beyond passive execution or adaption of assigned tasks, and they aim to prevent the reoccurrence of a problem (such as by addressing its root cause) or that involves solving it in an unusual and nonstandard way. It implies that employees need to develop their own goals, focus on long-term development, and adopt a long-term perspective to prevent problems (Frese and Fay, 2001). According to Renn et al. (2005), DG is a psychological factor with cognitive and motivational functions. It can promote individuals to take the initiative to work for better income and position in the future and to go upstream to realize self-development. Compared with individuals with low DG, employees with high DG will pay more attention to long-term performance rewards and future development, and strive to pursue more valuable and challenging goals in their work. Therefore, they would like to strengthen self-management and self-enhancement (Renn et al., 2005), and take the initiative to engage in future-oriented actions for performance improvement and self-enhance (Parker et al., 2019; Zhang et al., 2022). Overall, PAI has a positive but marginal decrease effect in DG, and DG is positively related to PWB. Accordingly, we propose the following hypothesis:

*Hypothesis 2*: DG is positively related to PWB and plays a positive mediating role between PAI and PWB.

### 2.2. The time-losses effect of PAI

#### 2.2.1. The relationship between PAI and PU

According to PM theory, in the performance process, the PM proved by time-related factors is rarely static and often changes, dissipates, or fluctuates over time (Briki et al., 2016; Briki and Markman, 2018), which is effect by the perceived probability of goal achievement (Iso-Ahola and Dotson, 2014). The events that influence goal attainment will produce negative PM and then cause individual emotional fluctuations and negative behavior patterns (Jones and Harwood, 2008; Iso-Ahola and Dotson, 2014). With the extension of the PAI, the increase of uncertainties and risk factors can decrease employees’ PM and make individuals negative and conservative. Aligning with these, we assumed that PAI is positively related to PU.

In detail, uncertainty refers to the degree to which the future states of the environment cannot be accurately anticipated or predicted, which has been proven to be a main factor to influence employees’ initiative (Bordia et al., 2004a; Hon et al., 2014). With the extension of PAI, the uncontrollable factors or unpredictable events within the interval prevent reward attainment increase (Bulley et al., 2016; Wang and He, 2020). Individuals cannot predict whether their initiative can get results, such as promotion opportunities, changes to the job role, or individual needs, increasing job-related uncertainty (Maurier and Northcott, 2000; Ito and Brotheridge, 2001). Besides, as individual needs and demands would be changing as time goes by, the perceived utility of further future rewards is uncertain (Scholten et al., 2019), which increases the uncertainty of the reward value. Meanwhile, the input–output chain is blurring gradually, leading to the stimulation of performance appraisal will wear off, which also increases the uncertainty within the interval. Therefore, we assume that PAI positively affects employees’ PU.

According to PM theory, contrary to positive PM, negative PM tends to increase marginally along with the development of the event (Briki and Markman, 2018), which is because individuals are more sensitive to negative events and are more likely to be affected by them (Jones and Harwood, 2008). Align with this, the extension of PAI is accompanied by an increased frequency of events with uncertainty, then leading to a positive and marginal increase relationship between PAI and UP. Accordingly, we propose the following hypotheses:

*Hypothesis 3*: PAI is positively related to PU, and the marginal effect is increasing.

#### 2.2.2. The mediation of PU between PAI and PWB

Uncertainties and risk factors can decrease employees’ PM and make individuals negative and conservative. According to PM theory, such negative PM triggered by PU can cause individual emotional fluctuations and negative behavior patterns (Jones and Harwood, 2008; Briki and Markman, 2018). Aligning with these, we assumed that PU is negatively related to PU. Uncertainty is the sense of doubt about future events about cause-and-effect relationships, and it is considered to be an aversive state (Schuler, 1980; DiFonzo and Bordia, 1998). With the extension of PAI and the increase of uncertainty, individuals gradually lack knowledge about future events and not knowing things about themselves or the environment, thus cannot prepare for the future or develop a future-orientation plan. Previous research indicated that uncertainty would undermine individuals’ initiative to influence or change events, and thus leads to negative consequences or behaviors, such as lead to low morale, lower performance, and even turnover (Bazerman, 1982; Jimmieson and Terry, 1999; Bordia et al., 2004b). Therefore, we predict that with the increase in PU, employees believe that the outcome and rewards of their proactive behaviors are ambiguous, thus they would be risk-averse and hold the performance-avoidance strategy (Ma et al., 2023). They no longer pursue performance rewards and thus reduce their PWB.

As PAI has a positive and marginal increase effect in PU, and PU is negatively related to PWB, we thus assume that PU has a mediation role on PAI and PWB. That is, as the extension of PAI, employees’ PU associated the work initiative with its delayed reward increase. The increased PU will reduce the perceived possibility of reward attainment, thus having a corrosive effect on the intrinsic motivation of performance appraisal and leading to a decrease in PWB. Accordingly, we propose the following hypothesis:

*Hypothesis 4*: PU is negatively related to PWB and plays a negative mediating role between PAI and PWB.

### 2.3. The temporal framing effect of PAI on PWB

According to Haans et al. (2016) research, if the two potential antagonistic mechanisms are essentially gain and loss in nature, the total effect can be calculated by algebraic summation and eventually, forms a nonlinear total effect, which is named the “additive benefit and cost.” In this study, The two mechanisms (time-gain effect and time-loss effect) of PAI have the nature of gain and loss. Therefore, we added the time-gain effect and time-loss effect together and except that there is an inverted U-shape relationship between PAI and PWB.

To explain, when PAI is short, extending PAI exerts a bigger positive effect on DG than PU. The increased PWB brought by DG is higher than the decreased part caused by PU. The time-gain effect is dominant, and the overall PWB is increasing. However, when PAI increases beyond a saturation point, extending PAI greatly intensifies the psychological risk of reward attainment. Individuals are more sensitive to loss than gain. Thus, the increased PWB brought by DG is lower than the decreased part caused by PU, the time-loss effect gradually exceeds the time-gain effect. At this time, individuals take loss avoidance thus appearing preference reversal, PWB overall decreasing trend.

*Hypothesis 5*: PAI has an inverted U-shape effect on PWB.

### 2.4. The moderating role of SDF

The correlation between PAI and PWB will be gradually blurring with the extension of PAI. As SDF involves future-based helpful information, valuable guidance, or planning, it can make employees more sensitive to interrelated time scales and thus help them to clear their performance goals (Zhou, 2003; Li et al., 2011), thus leading a stronger positive PM as well as a weaker negative PM. Align with this, we expect that SDF moderates the relationship between the PAI and PWB.

Specifically, previous research has pointed out that feedback, if provided with corresponding solutions, can bring stronger intrinsic motivation and PM for performance improvement (Kluger and Denisi, 1996; Iso-Ahola and Dotson, 2014). As such, SDF not only can help subordinates clarify the task time information and dynamics of performance progress, but also provide their work plan and guidance for the next phase of work. Benefiting from this, they can perceive a higher correlation between future rewards and present behavior as well as a higher possibility of performance goal attainment (Harrison and Dossinge, 2017). It serves as an incentive to inject them with positive PM to improve their tendency of delayed gratification (Spreitzer et al., 2005; Kim et al., 2020), then leading to a higher level of PWB. Besides this, future performance information involved in SDF can reduce subordinates’ fuzzy feeling about task information, thus enhancing their sense of control and perception of certainty in work (Harrison and Dossinge, 2017), which in turn alleviates their PU as well as the performance “short-sightedness” backfired by long PAI (Wang and Duan, 2021). Therefore, we propose that the above two factors can slow the marginal decreasing speed of the time-gain effect and alleviate the marginal increasing speed of the time-loss effect, leading to a fatter U-shape relationship between the PAI and PWB. Accordingly, we propose the following hypotheses:

*Hypothesis 6*: SDF moderates the inverted U-shape relationship between the PAI and PWB, that is, with higher levels of SDF, the relationship between PAI and DG is stronger and the relationship between PAI and PU is weaker, leading to a flatter inverted U-shape relationship between PAI and PWB.

## 3. Methods

### 3.1. Sample and procedure

We collect data from high-tech enterprises in Shanghai. On the one hand, the staffing structure of high-tech enterprises are concentrated in R&D and technical posts, and there are more teams involved in R&D work, a series of complex and highly autonomous task, or technical work contents, which requires highly for PWB (e.g., task revision, role innovation, or transcendent behavior). Meanwhile, such teams usually adopt a relatively long PAI. On the other hand, R&D and technical posts involve lots of complex and highly autonomous tasks and face more unknowns and uncertainties. SDF belongs to the information feedback mode, professional knowledge and skills contained in SDF as well as the future-based and development-based perspective from above superiors help them “think out of the Box” and enable them to develop and make improvements (Zhou, 2003; Zheng et al., 2020), thus are more urged for such posts (Zhou, 1998; Zhou, 2003). In addition, SDF is a supportive condition for R&D employees to break with routine and try new methods without being afraid of the risks associated with breakthrough and exploration (Su et al., 2019), which justifies the prominent importance of SDF for our respondents. Therefore, the selection of these samples facilitated us to investigate the relationship between PAI and PWB and the role of SDF in it.

This research is supported by a special project of the Shanghai Municipal Finance Bureau (SMFB). In detail, we obtained a list of 120 high-tech enterprises and their contact information from the Enterprise Division of SMFB. From the list, we selected companies with more than 100 people and no large-scale staff turnover in the past 6 months, because such companies are stable and have a more mature performance evaluation system. Further, we get in touch with the Human Resources Department of the selected companies and introduced the content, purpose, and non-public nature of our study to them. Eventually, 21 enterprises expressed their willingness to provide support for our research, involving digital technology applications (8), intelligent manufacturing (6), communication equipment (4), biotechnology (2), and new energy materials research and development (1). We surveyed with the help of the corporate HR department and initially identified a team-level sample of 63.

Our study was conducted by questionnaires and we collected data in two phases *via* online surveys hosted by WeChat, a popular messaging app in China. To reduce common method bias, the data of each employee was collected from dual sources—employees themselves and their supervisors. As for the variable PWB, we asked team supervisors to evaluate it to reduce the subjectivity of self-evaluated variables. Therefore, before the survey, we numbered each employee by way of a WeChat group nickname. Before the survey, we emphasized the purpose, anonymous, and confidential nature of the research process. In the first phase, we first send the questionnaires for team leaders in each WeChat group and asked the team leaders about the shortest reward-related PAI they are using and then, evaluate the PWB of their employees (team members). At the same time, we send the questionnaires A for employees to ask them to fill in their demographic information and report the items of DG and PU. In the second phase, which was about half a month later, we send questionnaire B for employees to ask them to evaluate the SDF of their team leaders. Finally, we matched the data with the same number and the entire investigation is strictly coded.

In the selected 21 companies, a total of 703 employee questionnaires from 63 groups were collected. The final valid sample was made up of 622 participants from 57 groups. The effective response rate was 86.17%. Of the 622 employees, 64.31% were males, and females accounted for 35.69%. In terms of age, the distribution is mainly under 30 years old (38.42%), followed by 31–40 years old (30.87%), 41–50 years old (19.45%), and over 51 years old (11.25%). Regarding educational levels, 40.03% have a bachelor’s degree, 39.39% earned a master’s degree, 11.09% have a high school diploma or below, and 9.49% earned a doctorate (Those who are studying for a master’s degree currently were counted as bachelors). Regarding the time working with leaders, most people have worked with their leaders for 1–3 years (35.05%), followed by 4–6 years (27.17%), 7–9 years (25.56%), and 10 years or more (12.22%).

### 3.2. Measures

#### 3.2.1. Performance appraisal interval

We used Zhang (2013) single item to measure PAI. The item is “According to the company’s appraisal system, how often does your group conduct a pay-for-performance related appraisal?.” There are six intervals (monthly, bimonthly, quarterly, half-yearly, yearly, and three-yearly), and it was filled in according to the actual situation.

#### 3.2.2. Delay of gratification

We measured this variable using an 8-item questionnaire developed by Liu et al. (2007). It contains both work delay of gratification (4-item) and vocational delay (4-item) dimensions. Sample items such as “I always put my things on the back burner and complete the assigned work immediately” and “I often work late into the night to get the job done better.” The Likert-type items range from 1 (strongly disagree) to 5 (strongly agree). The Cronbach’s Alpha of the scale in this study is 0.83.

#### 3.2.3. Perceived uncertainty

This variable was measured by 4 items selected from the scale developed by Bordia et al. (2004a). Their questionnaire consists of three dimensions, namely strategic uncertainty, structural uncertainty, and job uncertainty. According to the themes and needs of our study, we selected the 4 items of job uncertainty (e.g., I am not sure to what extent my job role and tasks will change; I’m not sure whether I need to do more for promotion). The Likert-type items range from 1 (strongly disagree) to 5 (strongly agree). The Cronbach’s Alpha of the scale in this study is 0.91.

#### 3.2.4. Proactive work behavior

We measured PWB with an 8-item questionnaire developed by Parker et al. (2006). The scale contains two dimensions: proactive implementation of ideas (4-item) and proactive problem solving (4-item). The sample items are “I will try to solve the problem until it does not happen again” and “I will try to find out why there’s a gradual decline in performance.” The Likert-type items range from 1 (strongly disagree) to 5 (strongly agree). The Cronbach’s Alpha of the scale in this study is 0.90.

#### 3.2.5. Supervisor developmental feedback

We used a 3-item scale adopted by Zhou (2003) to measure SDF. The sample items are “My supervisor provides me with useful information on how to improve my job performance” and “The purpose of feedback provided by my supervisor is to help me learn and improve.” The Likert-type items range from 1 (strongly disagree) to 5 (strongly agree). The Cronbach’s Alpha of the scale in this study is 0.97.

#### 3.2.6. Control variables

Previous research has shown gender, age, education (Erdogan and Bauer, 2005), and relationship duration (Grant et al., 2009) have an impact on proactive work behavior. Therefore, we controlled for gender (female = 0 and male = 1), age, levels of education of superior and subordinate respectively, and years of working with their supervisor in the analyses.

### 3.3. Analytical strategies

Due to our respondents being collected from different groups, we first need to construct a null model and calculate the ratio of inter-group variance and intra-group variance, assessing whether a cross-level analysis is needed. The ICC1 of DG, PU, SDF, and PWB were 0.26, 0.27, 0.22, and 0.31 respectively, and all of them meet the strong correlation degree suggested by James et al. (1984). Therefore, we conducted a multilevel confirmatory factor analysis (MCFA) and cross-level regression analysis on the model. Besides, SDF was a team-level variable, but to reduce self-evaluation bias and improve data authenticity, we asked each employee to evaluate the SDF of their team leaders, so we needed to test whether this data could be aggregated at the team level. The ICC2 of SDF is 0.78, more than the aggregability standard of 0.06. Therefore, SDF can be aggregated at the team level.

Following Qin et al. (2018) research, we performed grand-mean centering on the variables in level 2 and group-mean centering on the variables in level 1 to distinguish the variances between different levels. Referring to the path analysis technique suggested by Edwards and Lambert (2007) and the code provided by Preacher and Hayes (2008), we have written the program on Mplus 8.4 to perform hypothesis testing. Especially, as the PAI is an ordinal variable so we conducted a categorical regression and set PAI as an ordinal variable. Further, we used the Johnson-Neyman (J-N) technique proposed by Lind and Mehlum (2010) to plot the specific trend of the non-linear effects as well as its confidence bands.

## 4. Results

### 4.1. Preliminary analysis

#### 4.1.1. Confirmatory factor analysis

As PAI is a single-item variable, we thus conducted a multilevel MCFA to test the discriminative validity of the other four variables. We compared the hypothesized 4-factor model with a series of alternative models (see Table 1). The results showed that the hypothesized four-factor model had a better fit (*χ*^2^/df = 2.89, RMSEA = 0.05, SRM_(within)_ = 0.04, SRMR_(between)_ = 0.04, CFI = 0.91, NFI =0.88, IFI = 0.92) than the three-factor model (*χ*^2^/df = 5.32, RMSEA = 0.08, SRM_(within)_ = 0.05, SRMR_(between)_ = 0.08, CFI = 0.78, NFI =0.76, IFI = 0.79), the two-factor model (*χ*^2^/df = 9.72, RMSEA = 0.12, SRM_(within)_ = 0.13, SRMR_(between)_ = 0.37, CFI = 0.53, NFI = 0.53, IFI = 0.55), and the one-factor model (*χ*^2^/df = 2.62, RMSEA = 0.07, SRM_(within)_ = 0.03, SRMR_(between)_ = 0.04, CFI = 032, NFI = 0321, IFI = 0.84). These results indicated the good discriminant validity of our study model.

**Table 1:** Confirmatory factor analysis.

	*χ*^2^/df	df	RMSEA	SRMR		CFI	NFI	IFI
				Within	Between			
Four-Factor Model (SDF, DG, PU, PWB)	2.89**	37	0.05	0.04	0.04	0.92	0.88	0.92
Three-Factor Model (DG + SDF, PU, PWB)	5.32**	40	0.08	0.05	0.08	0.79	0.76	0.79
Two-Factor Model (DG + PU + SDF, PWB)	9.72**	43	0.12	0.13	0.37	0.55	0.53	0.55
One-Factor Model (DG + PU + SDF + PWB)	13.84**	44	0.14	0.53	0.38	0.32	0.31	0.32

N_level-1_ = 57, N_level-2_ = 622; Variables PU and PWB are parceled based on dimensions; ^**^p < 0.01. PAI, performance appraisal interval; DG, delay of gratification; PU, perceived uncertainty; PWB, proactive work behavior; SDF, supervisor developmental feedback.

#### 4.1.2. Descriptive statistics

Means, standard deviations of the study variables as well as their correlations were reported in Table 2. There is a positive correlation between PAI and DG (*r* = 0.20, *p* < 0.01), PAI and PU (*r* = 0.35, *p* < 0.01), and DG and PWB (*r* = 0.31, *p* < 0.001). There is a negative correlation between PU and PWB (*r* = −0.26, *p* < 0.01), which provides preliminary support for hypothesis testing.

**Table 2:** Correlations, descriptive statistics, and reliability for all variables.

	M	SD	1	2	3	4	5	6	7	8	9
**Level-1 (N**_**level-1**_ **= 622)**											
1. Gender	0.64	0.50	–								
2. Age	2.37	0.87	0.05	–							
3. Levels of education	2.55	0.65	−0.06	−0.16^*^	–						
4. Years of working with supervisor	2.08	1.32	−0.07	0.22^**^	−0.06	–					
5. PAI (before converged)	3.76	0.94	0.07	0.03	0.07	0.04	–				
6. DG	3.16	0.88	0.09^*^	0.04	0.09^*^	0.05	0.20^**^	(0.83)	–		
7. PU	3.68	1.44	−0.11 ^*^	−0.05	0.04	0.04	0.35^**^	−0.03	(0.91)	–	
8.PWB	3.32	0.87	0.07	0.02	0.11^*^	−0.02	−0.04	0.31^***^	−0.26^**^	(0.90)	–
9. SDF (before converged)	3.58	0.95	0.04	0.02	0.09^*^	0.07	0.09	0.18^**^	−0.20^**^	0.14^*^	(0.87)
**Level-2 (N**_**level-2**_ **= 57)**											
1. PAI	3.76	0.94	0.04	-							
2. SDF	3.58	0.95	−0.03	0.06							

^*^p < 0.05, ^**^p < 0.01, ^***^p < 0.001; two-tailed test, reliability on the diagonal. PAI, performance appraisal interval; DG, delay of gratification; PU, perceived uncertainty; PWB, proactive work behavior; SDF, supervisor developmental feedback.

### 4.2. Hypothesis testing

**Figure 2:**
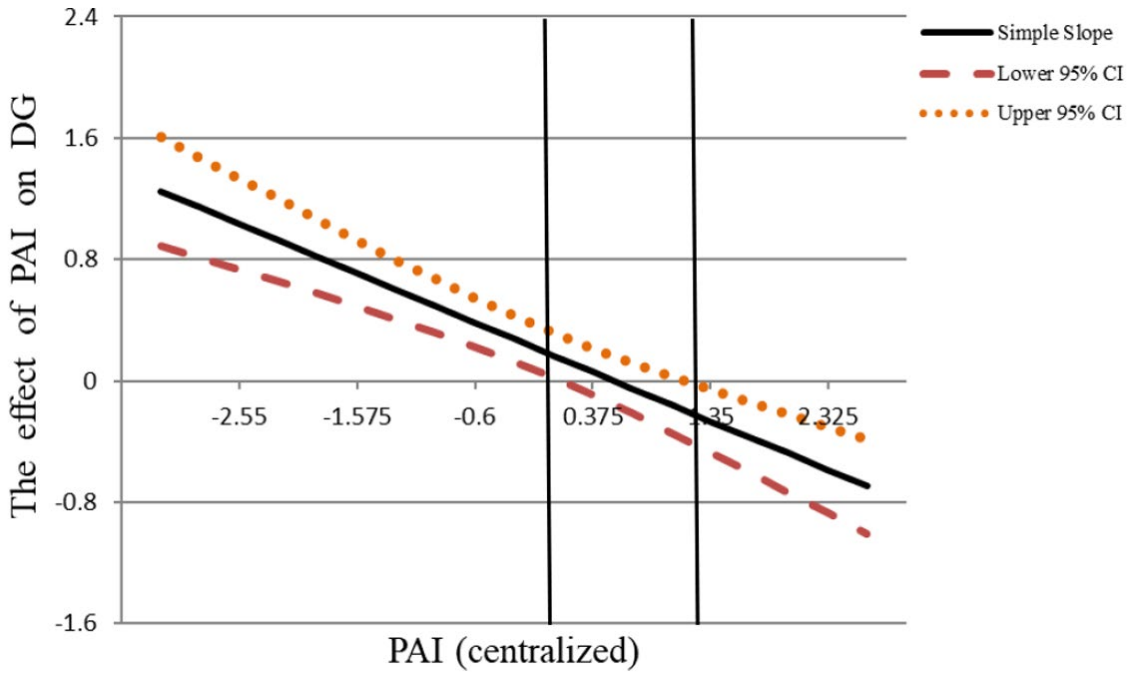
J-N diagram of PAI and DG. PAI, performance appraisal interval; DG, delay of gratification; PWB, proactive work behavior.

*Hypothesis 1* proposed that PAI had a positive but increasing marginal effect on DG. Model 1 in Table 3 shows that PAI was positively related to DG (*β* = 0.14，*p* < 0.01), and the quadratic term of PAI (PAI^2^) was negatively related to DG (*β* = −0.15，*p* < 0.01). The estimated slope at the minimum value (estimated slope = 1.30, *p* < 0.01) and maximum value (estimated slope = −0.64, *p* < 0.05) were also significant. The 95% CI [0.07, 0.80] of the turning point (PAI = 0.67) falls in its value range (−3.21, 2.65). That indicated that PAI has a positive effect at first but decreases continuously with the extension of the cycle. Further, we used Johnson-Neyman (J-N) diagram to visualize the curve shape. Figure 2 has shown that, when PAI is less than 0.08, the 95% CI of the simple slope is all above the X-axis, indicating that the simple slope estimate is significantly positive. Meanwhile, as PAI expands, the simple slope line slants to the lower right, and the simple slope estimate gradually decreases, indicating that the positive relationship between PAI and DG is marginally decreasing, and even becomes negative when PAI is greater than 1.2. *Hypothesis 1* was supported.

**Table 3:** Testing of the curve relationships between PAI, DG, PU, and PWB.

		Model 1 DG	Model 2 PU	Model 3 PWB
PAI	$\hat{\beta_{1}}$	0.14^**^ (0.05)	0.16^**^ (0.06)	0.05 (0.05)
PAI^2^	$\hat{\beta_{2}}$	−0.15^**^ (0.05)	0.08^*^ (0.04)	−0.14^**^ (0.05)
The slope at PAI min	$\hat{\beta_{1}}+2\hat{\beta_{2}}X_{L}$	1.30^**^ (0.41)	−0.07 (0.05)	0.46^**^ (0.14)
The slope at PAI max	$\hat{\beta_{1}}+2\hat{\beta_{2}}X_{H}$	−0.64^**^ (0.20)	0.80^**^ (0.23)	−0.33^*^ (0.13)
turning point	$\hat{\beta_{1}}/(-2\hat{\beta_{2}})$	0.67	−1.17	0.22
95% confidence interval		[0.07, 0.80]	[−0.57, −0.06]	[−0.26, 1.44]
X value range for PAI		[−3.21, 2.65]		

N_level-1_ = 57, N_level-2_ = 622; ^*^p < 0.05, ^**^p < 0.01; two-tailed test; PAI was centralized. PAI, performance appraisal interval; DG, delay of gratification; PU, perceived uncertainty; PWB, proactive work behavior.

**Figure 3:**
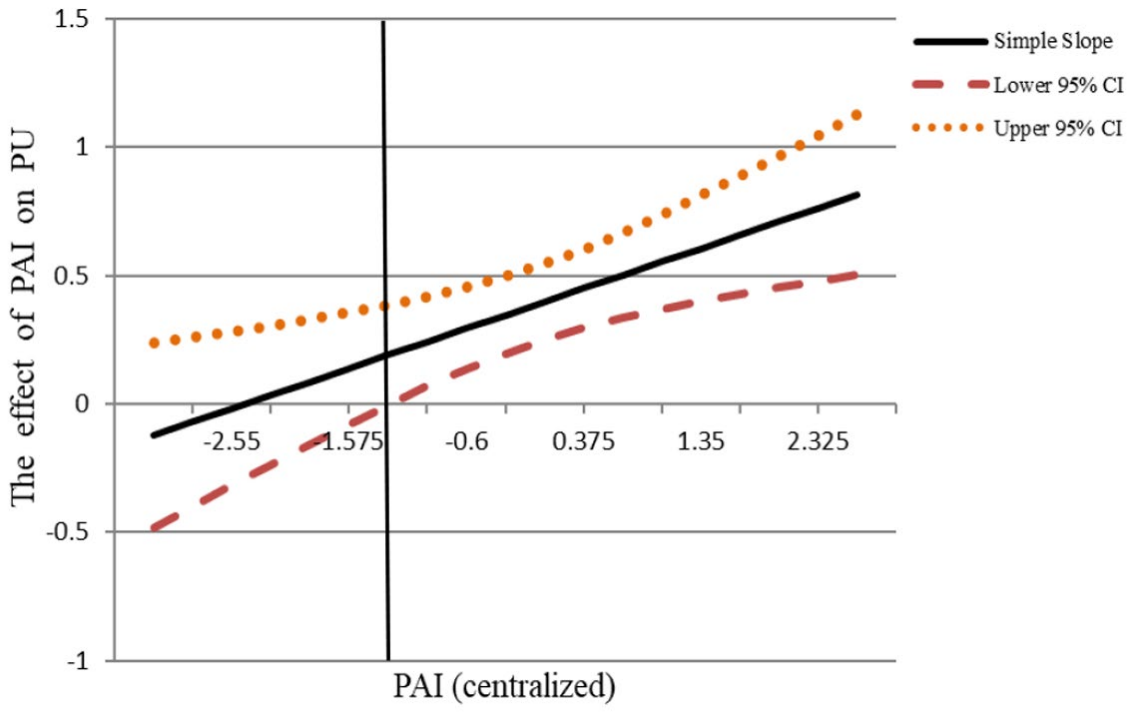
J-N diagram of PAI and PU. PAI, performance appraisal interval; PU, perceived uncertainty; PWB, proactive work behavior.

As such, *Hypothesis 3* proposed that PAI had a positive and diminishing marginal on PU. The M2 in Table 3 shows that PAI^2^ (*β* = 0.08, *p* < 0.05) is significantly related to PU, and the estimated slope at maximum PAI value is significant (*k* = 0.80, *p* < 0.01). Moreover, the upper limit of the 95% CI [−0.57, −0.06] of the turning point (PAI = −1.17) fell in the range of PAI. That all indicates that the simple slope of the latter part is positive with an increasing margin. Further, according to the J-N diagram (Figure 3), when the PAI is over - 1.23, the 95% CI is above the X-axis and the simple slope is increasing gradually, which indicates that when PAI is beyond - 1.23, the relationship between PAI and PU is positive and the positive relationship is marginal increase. Therefore, *Hypothesis 3* was well supported.

To test *Hypotheses 2* and *4*, Model 2 in Table 4 showed that DG positive related to PWB (*β* = 0.22, *p* < 0.01), and PAI^2^ positive related to PWB (*β* = 0.11, *p* < 0.01). Model 3 in Table 4 showed that PU was negatively related to PWB (*β* = −0.31, *p* < 0.01), and PAI^2^ was negatively related to PWB (*β* = −0.09, *p* < 0.05). Compared with Model 1, we know that DG and PU both play a partial mediating role. Therefore, *Hypothesis 2* and *Hypothesis 4* were supported.

**Table 4:** Regression analyses of proactive work behavior.

	PWB			
	M1	M2	M3	M4
Gender	0.03 (0.04)	−0.06 (0.05)	−0.05 (0.04)	0.09 (0.06)
Age	0.06 (0.05)	−0.03 (0.04)	0.05 (0.06)	0.08 (0.06)
Levels of education	0.17^*^ (0.07)	0.15^*^ (0.06)	0.17^*^(0.07)	0.12^*^ (0.05)
Years of working with supervisors	−0.04 (0.05)	−0.02 (0.07)	−0.01 (0.05)	−0.04 (0.05)
PAI	−0.07 (0.06)	0.07 (0.06)	−0.15^*^ (0.06)	−0.06 (0.06)
PAI^2^	−0.14^**^ (0.05)	−0.11^**^ (0.05)	−0.09^*^ (0.04)	−0.10^*^ (0.05)
DG		0.22^**^ (0.07)		
PU			−0.31^**^ (0.06)	
SDF				0.22^**^ (0.07)
PAI^2^*SDF				−0.15^*^ (0.06)
Within-group residual variance	0.36^**^	0.40^**^	0.32^**^	0.32^**^
Between-group residual variance	0.44^**^	0.31^**^	0.19^**^	0.34^**^

N_level − 1_ = 57, N_level-2_ = 622; ^*^p < 0.05, ^**^p < 0.01; PAI and SDF were centralized. PAI, performance appraisal interval; DG, delay of gratification; PU, perceived uncertainty; PWB, proactive work behavior; SDF, supervisor developmental feedback.

*Hypothesis 5* proposed that there would be an inverted U-shape relationship between PAI and PWB. Model 3 of Table 4 shows that the PAI^2^ was negatively related to PWB (*β* = −0.14, *p* < 0.01). Thus, *Hypothesis 5* was well supported.

**Figure 4:**
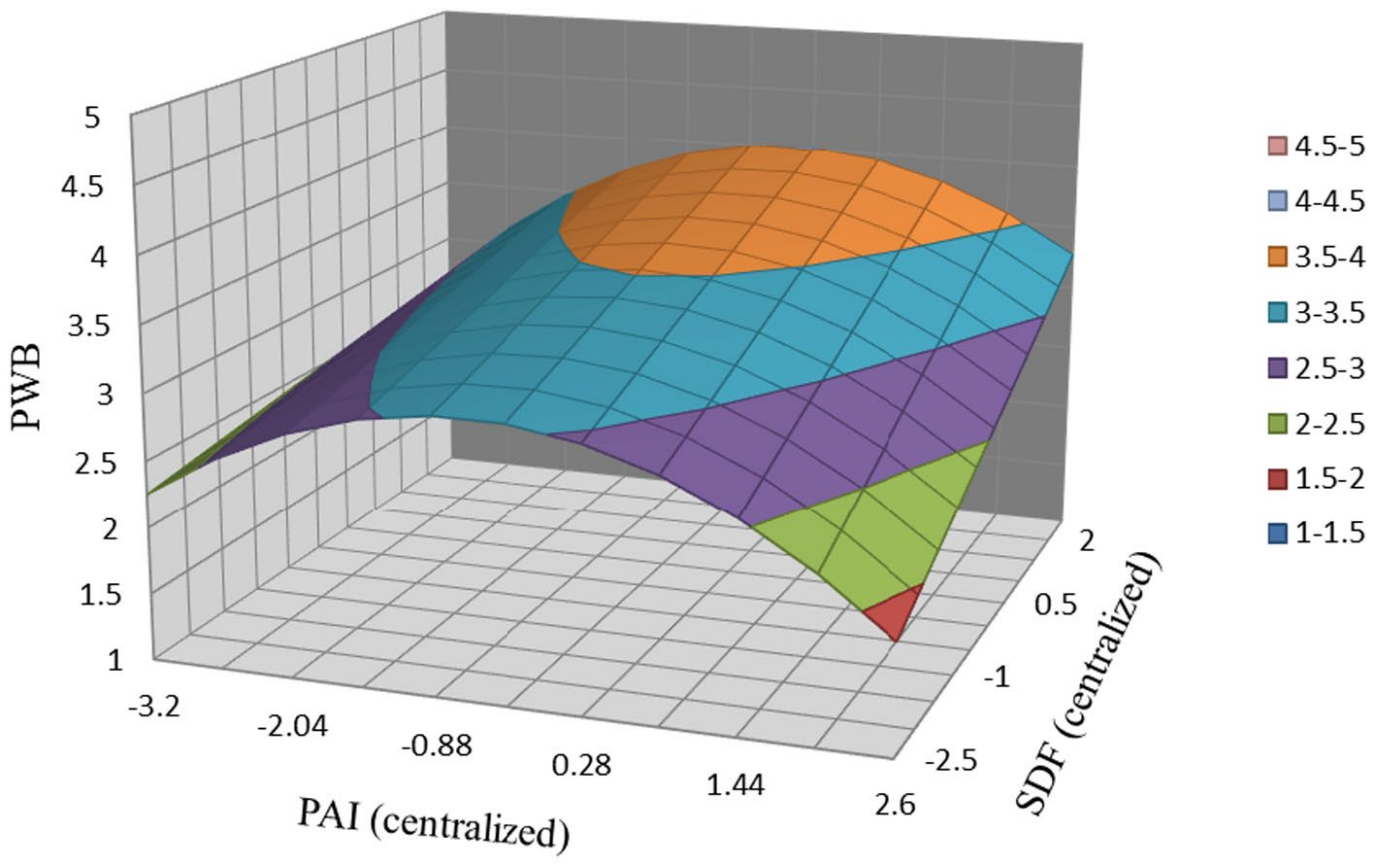
Three-dimensional Relationship between SDF, PAI, and PWB. PAI, performance appraisal interval; PWB, proactive work behavior; SDF, supervisor developmental feedback.

*Hypothesis 6* proposed that SDF moderates the inverted U-shape relationship between PAI and PWB. As shown in Model 4 of Table 4, the interaction term of PAI^2^ and SDF was positively related to PWB (*β* = −0.10, *p <* 0.05). Besides, Table 5 shows the simple slopes of PAI^2^ on PWB at the low (one SD below the mean) and high (one SD above the mean) levels of SDF. The results indicated that the simple slope was both significant whenever the SDF was low (slope estimate = −0.21, *p <* 0.01) or high (slope estimate = −0.12, *p <* 0.05), and the difference is significant (slope estimate = −0.09, *p <* 0.05), which indicated a flatter relationship with a high level of SDF. Therefore, *Hypothesis 6* was supported. To clarify the moderator path of SDF, we plotted the interaction effect in Figure 4. It can be seen that under the same PAI, the higher SDF, the higher PWB. Besides, SDF slow down the reduction of PWB in the second half of the curve and thus made the relationship flatter.

**Table 5:** Estimates of the simple slope and significance of the moderating effect.

			Simple slope	Standard error	*T* valve	*p* valve	95% Confidence interval	
							Lower	Upper
SDF	−1 SD	PAI	−0.09	0.05	−1.80	0.08	−0.19	0.01
		PAI^2^	−0.21^**^	0.07	−3.14	0.00	−0.35	−0.07
	+1 SD	PAI	−0.04	0.07	−0.57	0.56	−0.18	0.10
		PAI^2^	−0.12^*^	0.06	−2.00	0.04	−0.23	−0.01

*N* = 383; ^*^p < 0.05, ^**^p < 0.01; two-tailed test. PAI, performance appraisal interval; SDF, supervisor developmental feedback.

## 5. Discussion

### 5.1. Discussion of the results

Drawing upon the PM theory, we developed and tested a model explaining how PAI affects employees’ psychometric process of time gain and time loss and further improves their proactive work behavior. Findings from a multilevel survey of 622 employees from 57 groups revealed that PAI had an inverted U-shape effect on PWB.

With the “time” element gradually becoming sensitive and critical in the task situation (Tobin and Grondin, 2012; Stiglbauer, 2017), some researchers have begun to explore the influence of time factors on PWB, which focus on the influence of subjective temporal factors, such as time pressure, temporal categorization, and future orientation on PWB (Strauss et al., 2012; Tu and Soman, 2014; Urbach and Weigelt, 2019). Few empirical studies explore the relationship between organizations’ objective timeframe and PWB. According to Shipp and Cole (2015) research, “time is objective and subjective,” and the objective timeframe provides individuals an initial motivation for work proactivity, which indicates the importance to explore the relationship between organizations’ objective timeframe, such as PAI, and PWB. By focusing on the temporal framing effect of PAI, we changed from subjective temporal factors to objective organization timeframe and provide a new explanatory mechanism for stimulating PWB. Specifically, for the time-gain effect, PAI exerted a positive but marginal decreasing effect on employees’ DG, and then their PWB indirectly. For the time-loss effect, PAI exerted a positive and marginal increasing effect on employees’ PU and then decreased their PWB directly.

Further, according to the principle of “additive benefit and cost” proposed by Haans et al. (2016), we added the time-gain effect and time-loss effect together and found an inverted U-shape overall relationship between PAI and PWB. This finding indicated that a moderate PAI can increase employee future orientation and tendency of DG, and then promote employees’ PWB. But as the PAI extends to a certain way, the risk and uncertainty for performance attainment are amplified gradually and outweigh the time-gain effect, which results in the decrease of PWB. We assume that the inverted U-shaped impact is because: as mentioned above, the organization’s timeframe is an important consideration about whether afford employees to abandon more certain and passive activities and engage in PWB. When the PAI is narrative, extending PAI can increase the task time affluence and thus affords employees to put a lot of effort into performance improvement and self-development, the relationship between PAI and PWB is positive initially. However, with the continuing extension of the PAI, the time resource is gradually sufficiently becoming a slack resource (i.e., the pool of resources in an organization that is more than the minimum necessary to produce a given level of organizational output; Nohria and Gulati, 1997), thus time is no longer the considered factor for increasing the possibility of PWB, leading the time gain effect of extending PAI diminish gradually. Meanwhile, the increase of uncertainties and risk factors with the extension of PAI makes individuals less motivated and conservative (Hon et al., 2014), thus the relationship between PAI and PWB would decline. Our results verified Shipp and Cole (2015) reasoning that the effect trajectory of a time-related construct may be characterized as unstable, such as growth versus decline, and may exhibit predictable cycles. By exploring a more comprehensive explanation of how PAI acts on employees’ PWB, we found a compatible explanatory mechanism to integrate the positive and negative effects of changing PAI.

Moreover, our results verified that SDF has a moderation effect on the relationship between PAI and PWB. A higher level of SDF leads to a flatter inverted U-shape relationship between PAI and PWB. Specifically, in the rising part of the curve, when the SDF is higher, PAI has a more positive influence on PWB. On the descending part of the curve, higher SDF slow down the reduction of PWB and thus made the relationship flatter. By introducing such leadership variables as an important optimization strategy for enhancing the motivational effectiveness of PAI, this study offers a new pathway of how SDF influences employee PWB and has implications for motivating PWB in a relatively long-term PAI.

### 5.2. Theoretical implications

Our study has important theoretical implications. Firstly, we extend performance appraisal literature by shifting the predominant focus from performance indicator (Buckingham and Goodall, 2015; DeNisi and Murphy, 2017), performance rating (Murphy, 2020), performance equity (Leung et al., 2001), and performance feedback (Bracken et al., 2016; Murphy et al., 2018; Murphy, 2020) to performance appraisal interval as well as its temporal framing effect. Studies indicated that the “time” element becomes particularly sensitive and critical in the task situation (Tobin and Grondin, 2012; Stiglbauer, 2017; Parker et al., 2019), resulting that the time framing of task appraisal affects greatly the original incentive utility of organizational performance appraisal system. Therefore, PAI as well as its effects should be taken seriously, but few studies have considered it. Our research found that the PAI, by setting the timescale of performance tasks, exerts a mixed temporal framing effect on employees’ PWB. On the one hand, PAI has a time-gain effect. PAI exerted a positive but marginal decreasing effect on employees’ tendency of delayed gratification, leading to a higher level of PWB. On the other hand, PAI has a time-loss effect. PAI exerted a positive and marginal increasing effect on employees’ perceived uncertainty, then decreased the level of PWB. Overall, with the extension of PAI, the shift of the predominant factors will result in an inverted U-shape relationship between PAI and PWB. By highlighting the mixed temporal framing effect of PAI, we enrich the research of performance appraisal and clarify the incentive effect of PAI.

Secondly, by the addition of time-gain and time-loss PAI, we verified that PAI had an inverted U-shape effect on PWB. Our findings support this conclusion. According to the nonlinear causal principle proposed by Haans et al. (2016), we add the time-gain effect and time-loss effect together and found the inverted U-shape overall between PAI and PWB. That can be explained that while the extension of PAI has a positive effect on improving employees’ PWB initially, after PAI was extended beyond a saturation point, an inverse relationship exists, and the beneficial effect is swallowed by the perceived uncertainty, leading to an inverted relationship between PAI and PWB. In detail, when PAI is short, extending PAI exerts a bigger positive effect on DG than PU, the increased PWB brought by DG is higher than the decreased part caused by PU, the time-gain effect is dominant, and the overall PWB is increasing. However, when PAI increases beyond a saturation point, extending PAI greatly intensifies the psychological risk of reward attainment, and individuals are more sensitive to loss than gain. Thus, the increased PWB brought by DG is lower than the decreased part caused by PU, the time-loss effect gradually exceeds the time-gain effect, and individuals take loss avoidance thus appearing preference reversal, PWB overall decreasing trend. Our study found a compatible explanatory mechanism for the contradictory effects between PWB and PAI.

Finally, this study describes the moderation role of SDF in the temporal framing effect of PAI, thus improving the relationship between PAI and PWB. According to Murphy et al. (2018), feedback is one of the organizations’ flexible incentive strategies and can make up for the defects of rigid temporal mechanisms. From our result, SDF can not only help employees clarify the work demand, enhancing the correlation between future performance reward and current work behavior (Spreitzer et al., 2005), thus improving their tendency of delayed gratification, but also help them avoid uncertainty factors, reducing the performance “short-sightedness” the perceived uncertainty backfired by worrying about gain and loss (Harrison and Dossinge, 2017; Murphy et al., 2018). In this vein, it provides us a boundary to explore the temporal framing effect of PAI on employees’ PWB.

### 5.3. Management implications

This study also has important practical implications. First, organizations should pay attention to the temporal mechanism of performance appraisal. Currently, most organizations and companies focus performance appraisals on issues such as performance indicators, performance equity, performance feedback, or performance intensity. There is a very serious path dependence, such as annual rewards, when determining their PAI. According to our result, PAI has a complex and considerable effect on PWB. An appropriate extension of their appraisal timeframe is a time-based way to increase work proactivity. Therefore, organizations can increase employees’ future orientation and delayed gratification by moderately lengthening PAI, and leveraging the role of PAI, to increase employees’ PWB. However, while extending PAI can bring employees more time autonomy and stimulate their work initiative for self-development, it is undeniable that, as van Ruysseveldt and van Dijke (2011) stated, excessive temporal slack would reduce the timeliness of work-related information processing, which decreases employees’ executive power and a sense of urgency to develop proactive and effective work behaviors (van Ruysseveldt and van Dijke, 2011). This may back negatively affect PWB. Therefore, organizations cannot extend the PAI overly without considering the organization’s context and work characteristics.

Secondly, our study found that PAI has an inverted U-shape effect on PWB, which is the outcome of additive time-gain effect (positive) and time-loss effect (negative). Therefore, when organizations consider extending PAI to motivate such future-oriented and self-starting behaviors, they should try to manage the timing and duration of performance appraisals to maximize the time-gain effect effects and mitigate potential negative consequences brought by the time-loss effect. On the one hand, managers should consider employees’ DG as an important criterion in the recruitment process. Meanwhile, they should pay attention to the cultivation of employees’ career delayed gratification ability, and encourage employees to set up long-term career development goals and long-term work plans, instead of pursuing quick success. Besides, leaders should guide employees’ future development, leading them to pay more attention to long-term interests and future development, and then increase future-oriented behavior and initiative at work. Second, the importance of given future goals affects the degree of DG. When future goals are attractive, individuals’ motivation to follow long-term values is greater than their motivation for immediate gratification, and the motivation for DG increases. Therefore, when extending PAI, improving the given rewards of long-term performance appraisal may be considered. On the other hand, organizations and managers should set up an emergency warning system and emergency meetings to improve the ability to cope with uncertain situations. Leaders should try to reduce the uncertainties in the work process. For example, help employees to develop the overall time charter and task schedule for the overall appraisal cycle, provide specific guidance and planning for employees as a role of seniors, and provide solutions to work difficulties or bottlenecks encountered by employees. Which can reduce the weakening effect of uncertainty perception on PWB. Overall, by increasing the time-gain effect and decreasing the time-loss effect, making employees show more PWB within a certain interval.

Thirdly, leaders should focus on providing developmental feedback to their subordinates and improving the quality and guiding effect of feedback for the future. The performance appraisal system does clarify the organizational orientation and time tone, but not in every detail (Murphy et al., 2018). Therefore, periodic performance appraisal cannot replace leadership feedback, on the contrary, it needs developmental feedback as a flexible incentive strategy and useful supplement to make up for the defects of a rigid system (Murphy, 2020). In this regard, the content of leaders’ feedback should be related to employees’ personal growth and development as far as possible. For example, they should provide employees with vocational skills training and constructive information such as work performance improvement suggestions, and provide subordinates with valuable information to help them learn, develop and improve in the future, to meet the needs of employees’ development. This can mobilize employees’ enthusiasm for work so that they produce more positive work behavior.

## 6. Limitations and research directions

This study, inevitably, has some limitations. First, this study focuses on the high-tech industry. Future studies should focus on more industry types to obtain more wide-ranging results. Second, based on cross-sectional data collection, this study only focused on exploring the incentive utility of different PAIs, neglecting the fluctuation of PWB at different stages of a PAI. Therefore, future research should consider measuring employees’ PWB at several points within a PAI, which can decrease the common method bias and provide stronger evidence for the proposed relationships. Besides, multiple experience sampling can support us to construct a latent variable growth model and further explore the change of individual PWB along the development trajectory of PAI. Third, this study, from the leadership level, only examined one possible boundary condition of the relationship between PAI and PWB. Given that the work proactivity for future performance rewards is a matter of intertemporal decision-making, factors at the individual level (e.g., future work self; goal orientation; Strauss et al., 2012; Gerhart and Fang, 2015) play an important role in the progress. Future studies should consider the moderator at the individual level and construct a comprehensive perspective to understand the temporal framing effect of PAI. Finally, according to Pierce and Aguinis (2013), the curvilinear relationship is essentially a mechanism for reconciling trade-offs between two antagonistic relationships. Additive benefit and cost (Haans et al., 2016) and interactive motivation and ability are two mainly interactive modes of the potential mechanisms (Grant and Schwartz, 2011). In our study, we explain the inverted U-shape relationship between PAI and PWB according to the addition of the gain effect and loss effect. However, according to Blumberg and Pringle (1982) interactive theory of performance, the interaction of wiliness and ability can influence individuals’ behavior, and such factors can be changed be timeframe. Therefore, in future studies, it is worth exploring whether the interaction of some factors under a PAI can lead to this curvilinear relationship.

## 7. Conclusion

This study explored the time-gain effect and the time-loss effect of PAI on PWB as well as their boundary conditions. All hypotheses were supported by the findings. The results showed that: (1) PAI exerted a positive but marginal decreasing effect on delay of gratification (DG), and then increase PWB indirectly (i.e., the time-gain effect). (2) PAI exerted a positive and marginal increasing effect on perceived uncertainty (PU), and then decrease PWB indirectly (i.e., the time-loss effect). (3) According to the additive principle of the benefit and cost proposed by Haans et al. (2016), the addition of the time-gain effect and time-loss effect leads to an inverted U-shape effect of PAI on PWB. (4) Supervisor developmental feedback (SDF) moderated the inverted U-shape effect of PAI on PWB. These results advances employee proactivity research from a perspective of organizations’ time mechanisms, and also provides a theoretical basis for leaders to adopt developmental feedback as an optimization strategy. However, this study focuses on the high-tech industry and this finding should be verify in more wide-ranging industry types. Second, this study only focused on exploring the incentive utility of different PAIs. Future research should consider measuring employees’ PWB at several points within a PAI and further explore the change of individual PWB along the development trajectory of PAI. Besides, as we explored one possible boundary condition from the leadership level, future studies should consider the moderator at the individual level and construct a comprehensive perspective to understand the temporal framing effect of PAI.

## Data availability statement

The raw data supporting the conclusions of this article will be made available by the authors, without undue reservation.

## Author contributions

JY: methodology, project administration, software, validation, visualization, writing – original draft and editing, data curation, and formal analysis. JM: conceptualization and supervision. LL: funding acquisition, resources, and writing – review. All authors contributed to the article and approved the submitted version.

## Funding

Project funded by the 71st batch of China postdoctoral science foundation (2022M712020). Project funded by the 2022 Youth Fund for Humanities and Social Sciences of the Ministry of Education (22YJC630057).

## Conflict of interest

The authors declare that the research was conducted in the absence of any commercial or financial relationships that could be construed as a potential conflict of interest.

## Publisher’s note

All claims expressed in this article are solely those of the authors and do not necessarily represent those of their affiliated organizations, or those of the publisher, the editors and the reviewers. Any product that may be evaluated in this article, or claim that may be made by its manufacturer, is not guaranteed or endorsed by the publisher.
